# Melatonin has favorable preventive effects on experimental chronic pancreatitis rat model

**DOI:** 10.3906/sag-2103-134

**Published:** 2021-10-21

**Authors:** Esra GÜZEL TANOĞLU, Alpaslan TANOĞLU, Makbule Çisel MERİÇÖZ AYDIN, Muhammed Fevzi ESEN

**Affiliations:** 1 Department of Molecular Biology and Genetics, Institution of Hamidiye Medical Sciences, University of Health Sciences Turkey, İstanbul Turkey; 2 Experimental Medicine Research and Application Center, University of Health Sciences Turkey, İstanbul Turkey; 3 Department of Gastroenterology, Sultan Abdulhamid Han Training and Research Hospital, University of Health Sciences Turkey, İstanbul Turkey; 4 Department of Pathology, Koç University Hospital, İstanbul Turkey; 5 Department of Health Information Systems, Institution of Hamidiye Medical Sciences, University of Health Sciences Turkey, İstanbul Turkey

**Keywords:** Chronic pancreatitis, rat, melatonin, endoplasmic reticulum, ABC transporter genes

## Abstract

**Background/aim:**

Currently, there is not any specific treatment for chronic pancreatitis (CP). It was aimed to investigate the effects of melatonin administration on endoplasmic reticulum (ER) stress, oxidative stress, fibrosis, biochemical and histopathological parameters, and *Abcc2,Abcc5, *and *Abcg2 *gene levels in an experimental rat CP model.

**Materials and methods:**

Forty rats were randomized into five groups: Sham, CP, CP+25 mg/kg melatonin, CP+50 mg/kg melatonin, and CP+placebo. In all rats, except the sham group, a model of chronic pancreatitis was accomplished with intraperitoneal caerulein administration. In treatment groups, melatonin was used as a therapeutic agent. Serum TGF-β, TNF-α, MDA and GPx levels were studied. Pancreatic tissues were evaluated histopathologically. The expression levels of α*Sma,IR1*α*,Perk,Abcc2,Abcc5*, and *Abcg2* genes were measured with the qRT-PCR.

**Results:**

Biochemical results of the melatonin groups exhibited favorable changes compared to the CP and placebo groups. **α** *Sma,IR1α,Perk* expression levels were significantly lower in the melatonin groups. The expression levels of *Abcc2, Abcc5*, and *Abcg2 *were significantly higher in the CP group compared to the sham group, and these gene levels were significantly lower in the melatonin groups compared to the CP group (p < 0.01, p < 0.05, p < 0.05, respectively).

**Conclusion:**

In light of these favorable positive results, melatonin may be a useful preventive agent in the course of CP.

## 1. Introduction

Chronic pancreatitis (CP) is a disease characterized by progressive damage to the exocrine and endocrine parenchyma of the pancreas and is usually induced by alcohol and tobacco use [1]. Genetic and environmental factors also cause CP. It reduces the patient quality of life, causes other systemic diseases and can also lead to pancreatic cancer [2-4].

As with many chronic diseases, the mechanism of chronic pancreatitis is complex. Oxidative stress, impaired autophagy, ER stress, activation of proinflammatory cytokines, and increased apoptosis/necrosis have all been outlined in the pathogenesis of chronic pancreatitis. CP therapy mainly involves symptomatic treatments such as pain relief and replacement therapy for exocrine and endocrine insufficiency. Currently, there is no globally accepted effective treatment method for the clinical picture of CP [2–4]. For this reason, there is a great need to find new agents to prevent and treat CP as a first step and to determine the molecular structure of this disease. Melatonin is a hormone secreted from the pineal gland, with antioxidant and powerful antiinflammatory properties. Melatonin, which is associated with strengthening immune defense and modulation of apoptosis, has various functions within the cell, such as protecting the organism from damage caused by toxic radicals [5]. 

Melatonin protects tissues directly by the destruction of reactive oxygen and nitrogen species or indirectly by supporting the expression of antioxidant enzymes such as catalase (CAT), superoxide dismutase (SOD), glutathione peroxidase (GPx), and glutathione reductase (GR) [6, 7]. In addition, melatonin reduces the production of proinflammatory cytokines such as interleukin 1β, interleukin 6 (IL-6), interleukin 22, and tumor necrosis factor α (TNFα) [8].

The ABC gene family are transmembrane proteins that are expressed in lung, heart, pancreas, liver, kidneys, blood-brain barrier and placenta [9]. They have important roles in the transport of drugs, antibiotics, and toxins across biological membranes [10]. ABC transporters strongly affect drug absorption and pharmacokinetics. The effect of multidrug resistant proteins (ABC multidrug resistance) in many diseases is not widely known. There is no study in the literature about the expression and importance of the ABC proteins *Abcg2, Abcc2*, and *Abcc5* in the pancreas in an experimental CP model. There are articles showing the positive effects of melatonin in an experimental acute pancreatitis model, but the effects of melatonin in the experimental CP model was examined for the first time in this current study. In this study, the aim was to investigate the effects of melatonin administration on endoplasmic reticulum (ER) stress, oxidative stress, fibrosis, biochemical, and histopathological parameters and *Abcc2*, *Abcc5*, and *Abcg2* gene expression levels in an experimental rat chronic pancreatitis (CP) model.

## 2. Materials and methods

### 2.1. Rat groups

This research was conducted with the approval of the University of Health Sciences, Hamidiye Animal Experiments Local Ethics Committee, Turkey and the study was carried out in the University of Health Sciences Animal Experiments Center. Forty adult 16-20-week-old male Sprague Dawley rats weighing 250–350 g were used. Rats were housed in cages at approximately 24 °C. Food intake of animals, was stopped 12 h before sacrifice. Rats were randomized into five equal groups:

**Group 1:** Sham group (1 cc of saline was injected intraperitoneally (ip) twice a day at 09:00 a.m. and 10:00 a.m for 6 weeks, 3 days per week (Monday, Wednesday, Friday), and no other medication was administered).

**Group 2:** CP group (50 mcg/kg caerulein was injected ip twice a day (at 09:00 a.m. and 10:00 a.m) for 6 weeks, 3 days per a week. 

**Group 3:** CP+melatonin 25 mg/kg group (caerulein injections were performed as Group 2, melatonin was administered at a dose of 25 mg/kg ip, 3 days per a week for 6 weeks, 1 h before the caerulein injections, beginning from the first week of the study).

**Group 4:** CP+melatonin 50 mg/kg group (caerulein injections were performed as Group 2, melatonin was administered at a dose of 50 mg/kg ip, 3 days per a week for 6 weeks, 1 h before the caerulein injections, beginning from the first week of the study).

**Group 5:** Placebo group (caerulein injections were performed as Group 2, saline was injected IP at a dose of 1 cc ip, 3 days per a week for 6 weeks, 1 h before the caerulein injections, beginning from the first week of the study).

All rats were sacrificed 24 h after the last injections. Under general anesthesia, the rats were sacrificed by taking blood samples from the right atrium. The study was terminated after the pancreatic tissue samples were obtained. All blood samples were centrifuged at 5000 rpm for 10 min and then stored at –80 °C until biochemical analysis.

### 2.2. Biochemical analysis

**TGF-β, TNF-α, GPx, MDA: **Levels of TGF-β (transforming growth factor-beta), TNF-α, GPx and MDA (malondialdehyde) were tested with ELISA kits [Bioassay Technology Laboratory (BT Lab). Antibodies specific to rat TGF-β were placed in cavities within the Microtiter strips, followed by the addition of a second biotinylated antibody inside the wells. Serum samples were placed inside the wells using a pipette. All tests were carried out according to the instructor’s protocol.

### 2.3. Histopathological evaluation

Histopathological evaluation was made to evaluate pancreatic damage. After the rats were sacrificed, the pancreatic tissue was fixed in 10% buffered formaldehyde and then embedded in paraffin. Then, 3 μm thick sections were made from the paraffin blocks and hematoxylin & eosin staining was performed. Evaluation of pancreatic damage was performed by a blind pathologist. All samples were examined for inflammation, atrophy, and fibrosis. Correspondence analysis was performed to determine which histopathological scores were more common in which group.

### 2.4. Molecular methods

RNA isolation from pancreatic tissue samples: RNA isolation was performed from paraffinized pancreas tissues of rats with MasterPure Complete DNA and RNA purification kit (Lucigen, USA) according to the manufacturer’s protocol. RNA concentration and purities were measured with the aid of a Denovix DS-11 (DeNovix Inc., Wilmington, DE) spectrophotometer.

**cDNA synthesis and real time PCR (qRT-PCR): **Expression levels of *Abcc2, Abcc5*, and *Abcg2* genes and expression levels of *a-SMA *(in order to evaluate fibrosis development), *Ire1a *and *Perk* (in order to evaluate endoplasmic reticulum stress) genes were measured in all RNA samples obtained from rat pancreatic tissues**. **CDNA synthesis was completed using 500 ng of RNA. The cDNA synthesis was performed according to the manufacturer’s protocol using the Roche Transcriptor High Fidelity cDNA Synthesis Kit, Roche (Switzerland). *Gapdh* was used as an internal control. The samples were studied in duplicate with SYBR Green Master Mix from Roche (Switzerland) on the LightCycler 480 instrument. SYBR Green qRT-PCR was performed at 1 cycle of 95 °C for 5 min followed by 40 cycles of 95 °C for 10 s and 60 °C for 1 mins. Melting curve analysis was performed at temperatures between 65 ℃ and 95 ℃. Primer sequences used for qRT-PCR are given in Table 1.

**Table 1:** Primer list of all genes which were evaluated in this current study.

Gene Name	Forward	Reverse
Abcc2	CTGGTTGGAAACTTGGTCG	CAACTGCCACAATGTTGGT
Abcc5	AACAGGAAGGATTCTCAACAGG	TGAATGCTGGACGTGATATGG
Abcg2	AGTCCGGAAAACAGCTGAGA	CCCATCACAACGTCATCTTG
Ire1α	CCTGAGGAATTACTGGCTTCTC	TCCAGCATCTTGGTGGATG
Perk	CGCTGCTGCTGCTGTTCCTG	GCAATGCCTCGGCGTCTTCC
α-sma	TTCCAGCCTTCCTTTATCG	TTGGCGTACAGGTCCTTC
Gapdh	TATCGGACGCCTGGTTAC	CTGTGCCGTTGAACTTGC

Statistical analysis: IBM SPSS Statistics 20 software and InStat3 GraphPad Statistics Software (trial version) were used for all statistical analyses. P < 0.05 was considered statistically significant. The significance between group means ± standard deviation (SD) was analyzed by Tukey’s test following one-way ANOVA. Relative quantitation analysis of the results of real-time PCR experiments was performed using the delta-delta-CT method. The chi-square test was used to determine the variation in histopathologic scores according to group. 

## 3. Results

### 3.1. Biochemical results

When the study groups are examined in terms of TNF-α levels, there were significant differences between the CP group, placebo group and the low-dose and high-dose treatment groups (p < 0.01). When rat groups were examined in terms of TGF-β levels, again, there were significant differences between the CP group, placebo group and the low-dose and high-dose treatment groups (p < 0.01). There was a significant difference in MDA levels between the CP group and treatment groups (p < 0.001). Finally, there was a significant difference in GPx levels between the CP group and treatment groups (p < 0.001). In other words, melatonin treatment significantly improved oxidative stress in our experimental rat CP model (Table 2).

**Table 2:** Mean results and p values of biochemical parameters.

	Group 1 (CP)	Group 2(LD)	Group 3(HD)	Group 4(P)	Group 5(S)	p
GPx (ng/ml)	9.9 ± 3.7	13.01 ± 4.1	16.20 ± 3.9	9.66 ± 4.6	20.2 ± 5.2	p < 0.01*
TNF-alpha (ng/ml)	68.76 ± 9.2	50.35 ± 5.1	42.25 ± 6.6	61.13 ± 5.8	35.97 ± 7.4	p < 0.01*
MDA (nmol/ml)	2.39 ± 0.3	1.32 ± 0.2	1.01 ± 0.1	2.33 ± 0.4	0.8 ± 0.2	p < 0.001**
TGF-β (ng/L)	266.23 ± 15.9	194.7 ± 14.6	168.96 ± 16.6	259.8 ± 13.2	150.28 ± 9.1	p < 0.01*

One Way ANOVA test was used. Data is given as mean ± SD. CP: Chronic pancreatitis group, LD: Low dose melatonin group, HD: High dose melatonin group, P :Placebo group, S: Sham group, GPx:Glutathione peroxidase, TNF-alpha: Tumor necrosis factor-alpha, MDA: Malondialdehyde, TGF-β:Transforming growth factor *p < 0.01, **p < 0.001.

### 3.2. Histopathological examination results

**Figure 1:**
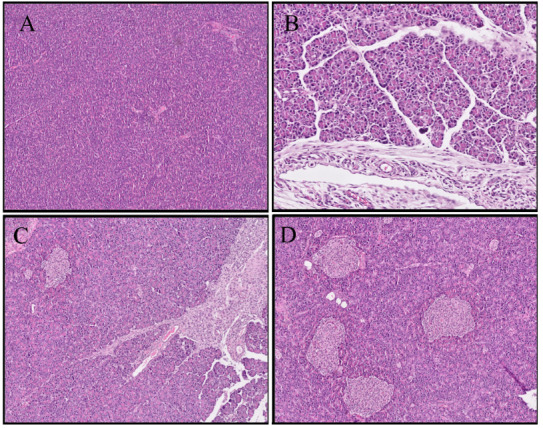
Hematoxylin and eosin (H&E) staining of pacreatic tissue samples. (a) Sham group, (pancreatic tissue with normal configuration and acinar structure) HE ×100, (b) chronic pancreatitis group, (dense inflammation, edema and glandular atrophy in pancreatic acinar structures with adjacent fibrosis) HE ×200, (c) low dose melatonin group (mild inflammation and minimal fibrosis in periductal area) HE ×100, (d) high dose melatonin group (minimal to mild inflammation in pancreatic tissue) HE ×200.

The histopathological scores were lower in the melatonin treatment groups than in the CP and placebo groups. The histopathological views of the experimental chronic pancreatitis model are shown in Figure 1. (1a-Sham group, 1b-chronic pancreatitis group, 1c-low dose melatonin group, 1d-high dose melatonin group)

There was no significant difference in histopathological score between the low-dose melatonin group and the CP and placebo groups (p > 0.05). There was a significant difference between the high-dose melatonin group and the CP and placebo groups (p < 0.05). Distribution of histopathologic scores by groups were given in Table 3.

**Table 3:** Distribution of histopathological scores by groups.

		Histopathological Scores				Total
		None	Mild	Moderate	Severe	
Groups	Low-dose	0	3	4	1	8
	CP	0	1	3	4	8
	Placebo	0	1	4	3	8
	Sham	8	0	0	0	8
	High-dose	1	4	3	0	8
Total		9	9	14	8	40

Note: A correspondence analysis was applied to determine which histopathological scores appeared more in which group. The difference between the high-dose group and CP and placebo group was significant (p < 0.05).

### 3.3. Molecular results

**Figure 2:**
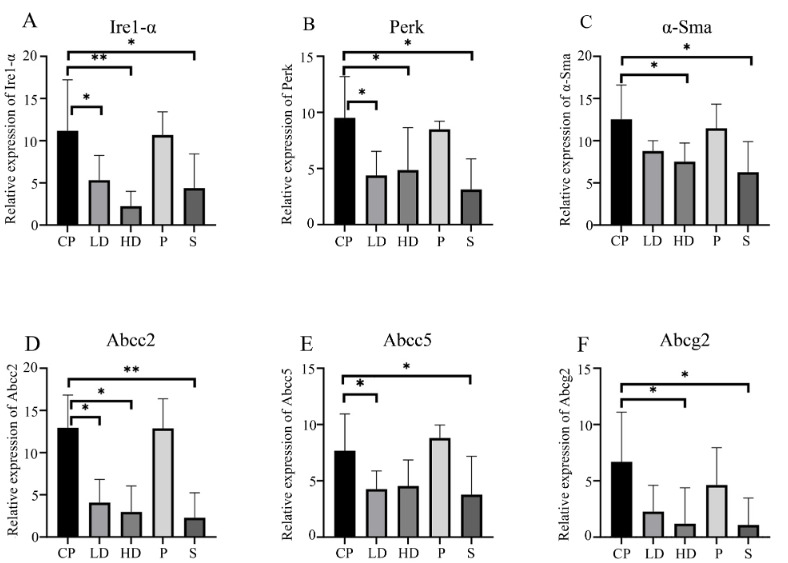
Expression levels of (a)IRE1a, (b)Perk, (c)a-Sma, (d)Abcc2, (e)Abcc5, and (f) Abcg2 in CP compared with the treatment groups and control. Data are expressed as the relative fold change among data sets, and each was performed in triplicate. CP: chronic pancreatitis, S: sham, HD: high dose melatonin, LD: low dose melatonin. One-way ANOVA with Tukey’s post-hoc test was used *p < 0.05, **p < 0.01.

The expression values of *a-Sma *(for fibrosis), *Ire1a and Perk *(for endoplasmic reticulum stress), and *Abcc2, Abcc5, *and *Abcg2* genes were determined by qRT-PCR in the pancreatic tissues of rats. The expression levels of all genes exhibited significant changes between the CP and sham groups (*Ire1a *p=0.03*, Perk *p=0.01*, a-Sma *p=0.01, *Abcc2 *p = 0.006*, Abcc5* p = 0.04 and *Abcg2 *p = 0.01). The expression levels of *Ire1a, Perk, a* *-Sma *were lower in the high-dose melatonin group compared to the CP group (p < 0.01, p < 0.05, p < 0.05, respectively) (Figure 2a, 2b, 2c). The expression levels of *Abcc2, Abcc5* and *Abcg2* genes were higher in the CP group compared to sham groups (p < 0.01, p < 0.05, p < 0.05, respectively). When the expression levels of *Abcc2, Abcc5* and *Abcg2 *genes were compared, *Abcc2 *and *Abcg2 *genes had lower expression levels in the high-dose group compared to the low-dose group (for all genes, p < 0.05) (Figures 2d–2f). For *Abcc5*, there was no significant difference in the expression levels of the high-dose melatonin and CP groups (Figure 2e). When the low-dose melatonin and CP groups were examined in terms of *Abcg2 *gene expression levels, we found no significant difference (p > 0.05) (Figure 2f). *Abcc2* expression level was significantly down regulated for low- and high-dose melatonin group compared to CP groups (p < 0.05) (Figure 2d). 

## 4. Discussion

Chronic pancreatitis is an inflammatory process in the exocrine and endocrine glandular pancreatic parenchyma resulting in progressive and irreversible damage and fibrosis. Currently, there is no single therapeutic agent for the etiopathogenesis of chronic pancreatitis, a disease with high morbidity rates [11]. This current research is the first study in the literature that examines the efficacy of melatonin in preventing the development of chronic pancreatitis and the effect of drug resistance genes at the histopathological, biochemical, and molecular levels in an experimental chronic pancreatitis model.

There are many methods that enable the induction of an experimental chronic pancreatitis model. One of these common applications is the model created with repeated caerulein injections [4, 12]. In previous studies, caerulein was shown to trigger cell necrosis by inhibition of the Wnt / β-Catenin pathway and lead to increased inflammatory cytokines. In-vivo studies showed that caerulein injection causes edema, inflammation, and necrosis in pancreatic acinar cells [13, 14].

Although recent studies provide us with a better understanding of the progression of chronic pancreatitis, there are still unclear points in the physiopathology. However, considering the multifactorial disease and complex clinical picture, the treatment of patients with CP is difficult and the applied methods result in failure in terms of treatment. In the literature, it was reported that the inhibitory effect of melatonin on the immune response is related to the antioxidant property of the molecule and that neutrophil activation prevents oxidant damage in tissues [15]. The absence of melatonin toxicity also supports that this agent can be used safely in many inflammatory processes like CP.

Although the efficacy of melatonin in the experimental CP model was not investigated in the literature, it was shown to have beneficial effects in many experimental acute pancreatitis models, and it is effective in reducing pancreatic damage and oxidative stress. Additionally, it was reported to reduce the severity of acute pancreatitis [16]. Melatonin was reported to regulate insulin secretion and has a protective effect on endoplasmic reticulum stress in an experimental acute pancreatitis model [17]. Melatonin protects against acute pancreatitis-associated pancreatic injury by down-regulation of IRE1α-mediated JNK/NF-κB pathways in rats [18]. Previously, Yildirim et al. performed an experimental rat CP study and used pentoxifylline as a therapeutic agent [13]. They showed that pentoxifylline decreased the inflammatory response and oxidative stress. In other words, their therapeutic agent had preventive efficacy in an experimental CP model. Similarly, in this current experimental CP model, we showed that melatonin ameliorated inflammatory response, oxidative stress, and endoplasmic reticulum stress.

The varying expression levels of ABC transporter genes are responsible for drug resistance in many chronic diseases and cancer [19]. Up-regulation of many kind of ABC transporters, including *ABCB1* and *ABCG2* leads to diminished cellular drug efflux [20]. Compatible with this fact, in this current study, we found significantly changed expression levels of ABC genes in the CP group. For the first time in the literature, in this current study, the expression levels of *Abcg2, Abcc5*, and *Abcg2* genes were investigated in an experimental CP model in which melatonin was used as a therapeutic agent.

In this current study, it has been shown that melatonin has positive protective properties, ameliorates inflammation, oxidative stress and pancreatic fibrosis. Moreover, it affects *Ire1a, Perk, a-Sma*, *Abcc2, Abcc5,* and *Abcg2* gene expression levels in an experimental CP model. In other words, melatonin may be used as a preventive and therapeutic option for CP in the future.

## Informed consent

The experimental protocol was approved by the University of Health Sciences, Hamidiye Animal Experiments Local Ethics Committee, Turkey (Approval Number: 2018-03/08)
